# Cerebellar-Cortical Connectivity Is Linked to Social Cognition Trans-Diagnostically

**DOI:** 10.3389/fpsyt.2020.573002

**Published:** 2020-11-04

**Authors:** Roscoe O. Brady, Adam Beermann, Madelaine Nye, Shaun M. Eack, Raquelle Mesholam-Gately, Matcheri S. Keshavan, Kathryn E. Lewandowski

**Affiliations:** ^1^Department of Psychiatry, Beth Israel Deaconess Medical Center, Boston, MA, United States; ^2^Department of Psychiatry, Harvard Medical School, Boston, MA, United States; ^3^Schizophrenia and Bipolar Disorder Program, McLean Hospital, Belmont, MA, United States; ^4^School of Social Work and Department of Psychiatry, University of Pittsburgh, Pittsburgh, PA, United States

**Keywords:** bipolar disorder, schizophrenia, psychosis, connectivity, social cognition, cerebellum, imaging, resting state

## Abstract

**Background:** Psychotic disorders are characterized by impairment in social cognitive processing, which is associated with poorer community functioning. However, the neural mechanisms of social impairment in psychosis remain unclear. Social impairment is a hallmark of other psychiatric illnesses as well, including autism spectrum disorders (ASD), and the nature and degree of social cognitive impairments across psychotic disorders and ASD are similar, suggesting that mechanisms that are known to underpin social impairments in ASD may also play a role in the impairments seen in psychosis. Specifically, in both humans and animal models of ASD, a cerebellar–parietal network has been identified that is directly related to social cognition and social functioning. In this study we examined social cognition and resting-state brain connectivity in people with psychosis and in neurotypical adults. We hypothesized that social cognition would be most strongly associated with cerebellar–parietal connectivity, even when using a whole-brain data driven approach.

**Methods:** We examined associations between brain connectivity and social cognition in a trans-diagnostic sample of people with psychosis (*n* = 81) and neurotypical controls (*n* = 45). Social cognition was assessed using the social cognition domain score of the MATRICS Consensus Cognitive Battery. We used a multivariate pattern analysis to correlate social cognition with resting-state functional connectivity at the individual voxel level.

**Results:** This approach identified a circuit between right cerebellar Crus I, II and left parietal cortex as the strongest correlate of social cognitive performance. This connectivity-cognition result was observed in both people with psychotic disorders and in neurotypical adults.

**Conclusions:** Using a data-driven whole brain approach we identified a cerebellar–parietal circuit that was robustly associated with social cognitive ability, consistent with findings from people with ASD and animal models. These findings suggest that this circuit may be marker of social cognitive impairment trans-diagnostically and support cerebellar–parietal connectivity as a potential therapeutic target for enhancing social cognition.

## Introduction

Psychotic disorders such as schizophrenia (SZ) spectrum disorders and bipolar disorder (BD) with psychosis are characterized by substantial impairment in social cognitive processing (1–3), which is associated with poorer community functioning (4–7). Social cognitive impairments have been reported in people with SZ and BD across multiple domains including various aspects of emotion processing such as facial affect recognition and “higher level” emotional reasoning (8–12), theory of mind (8, 13–15) and attributional style (16–18). Some aspects of social cognition appear to be more severely impaired in people with SZ compared to those with BD including “higher level” emotion processing (19, 20), theory of mind, and attributional style (10, 21), although in general differences appear more quantitative than qualitative, and empirical methods such as cluster analysis have revealed subgroups of patients cross-diagnostically who share similar levels of social cognitive functioning ranging from intact to more severely impaired (11).

Social cognitive impairments are not unique to psychosis but represent hallmark symptoms in other psychiatric disorders as well, including autism spectrum disorders (ASD). Recent evidence indicates that, behaviorally, people with psychotic disorders and ASD exhibit similar widespread social cognitive impairment relative to controls (22–24). Thus, it is possible that neural mechanisms believed to underpin social cognitive impairment in ASD may offer clues to neural substrates underlying similar deficits in SZ and BD. However, the extent to which similar behavioral phenotypes are underpinned by common neurobiological mechanisms across diagnoses is unclear.

Trans-diagnostic studies of neuroimaging and social cognitive impairment in people with SZ-spectrum disorders and ASD participants have been mixed, with some showing similar activation or connectivity patterns and others showing only partial overlap. In studies using fMRI-measured task-based activation, reduced frontolimbic and superior temporal sulcus (STS) engagement during social cognition tasks was a shared feature across diagnoses (25–27). Cortical connectivity abnormalities in default mode network (DMN) and salience networks were also common to both SZ-spectrum and ASD in adults and adolescents (28, 29) which were associated with abnormalities during mentalizing (29) and associated with severity of social impairment (30). However, some findings report diagnostic differences in regional activation even when task performance is similar (31) suggesting that similar behavioral phenotypes may result from different underlying mechanisms. Similarly, the above meta-analysis (27) found diagnosis-specific activation abnormalities including reduced thalamic and amygdala activation and ventrolateral prefrontal dysfunction primarily in SZ, decreased somatosensory engagement in ASD, and some task-specific differences in activation patterns. Overall, these findings suggest that some regional activation and network connectivity abnormalities may be common trans-diagnostically and associated with social cognition and functioning, although no clear mechanistic pathway has been identified within or across disorders. These studies have largely been based on purely correlational experiments, however, making it difficult to determine whether these associations are causal or are reflective of diagnosis-related epiphenomena.

While much work on the neurobiology of social cognition in psychosis has focused on cortical and limbic activation and connectivity (3, 32, 33), abnormalities of the cerebellum have consistently been reported in psychiatric disorders characterized by social cognitive impairments including SZ (34–38) and ASD (39–42). While cerebellum is commonly considered in terms of motor behavior, the cerebellum appears to play an important role in social cognition and emotion processing [e.g., (43–46)] and may be associated with social and emotional processing impairments seen in psychotic disorders and ASD. Few empirical reports have linked cerebellar abnormalities to social cognitive impairments in SZ [see (47)], and there are no such reports we are aware of in BD. However, recent evidence of associations between cerebellum and social cognition in ASD provide evidence of a specific cerebellar-cortical circuit directly related to social cognition.

Stoodley et al. used neuromodulation in humans and mice to demonstrate a *causal* association between connectivity of the Right Crus I (R Crus I) region of the cerebellum (commonly implicated in ASD) and the inferior parietal lobule and social behavior (48). Using neuromodulation, they identified a cerebellar–parietal circuit in neurotypical humans, and abnormalities of functional connectivity in this same circuit in children with ASD. They then went on to demonstrate that chemogenetically mediated inhibition of R Crus I activity in mice produced social behavioral impairment, whereas stimulation of R Crus I in a transgenic ASD mouse model rescued aberrant social behaviors. These novel findings are consistent with previous evidence from lesion studies in humans and animal models (49, 50), and suggest that this cerebellar parietal circuit may be directly and causally associated with social cognition in ASD. Whether this circuit is associated with social cognition in humans with psychotic disorders and thereby represents a trans-diagnostic mechanism for social processing impairments remains unknown.

In this report we aimed to examine whether previous findings of social cognition-connectivity associations in ASD were also present in people with psychotic disorders including SZ and BD. Specifically, we examined social cognition in association with resting-state (rsfMRI) brain connectivity in a trans-diagnostic sample of people with psychotic disorders as well as neurotypical controls using a data-driven, whole brain approach. We hypothesized that (1) people with psychosis would perform worse than neurotypical controls on an emotion management/emotion regulation task of social cognition; (2) social cognitive performance would be positively correlated with connectivity in the cerebellar–parietal circuit identified in people with ASD (48); and (3) associations between social cognitive performance and cerebellar–parietal connectivity would be similar across groups, indicating that this circuit is a common pathway underpinning social cognition.

## Materials and Methods

### Participants

Participants included people with a diagnosis of SZ or BD with psychosis (*n* = 81) and neurotypical controls (*n* = 45). Participants were recruited at three collaborating health centers via clinical programs including early psychosis specialty care, and through community referral networks, in the context of several separate research studies. Participants recruited from the Boston and Pittsburgh sites participated in a clinical trial (BICEPS, NCT01561859). Only the baseline (pre-intervention) evaluation data for these participants were included for this analysis. At the McLean site participants were recruited in the context of two separate but related studies including a study of cognitive remediation in bipolar disorder (TREC-BD, NCT01470781) and a study of clinical and cognitive characterization of psychosis. For subjects who participated in the cognitive remediation intervention study, only baseline cognitive and imaging data were included here. All procedures were approved by the Institutional Review Boards of the University of Pittsburgh (Pittsburgh, PA), McLean Hospital (Belmont, MA), and Beth Israel Deaconess Medical Center (Boston, MA). Every participant provided written informed consent prior to their participation. A subset of the data analyzed here was previously presented in Ling et al. (51).

Across sites, diagnosis was determined using the Structured Clinical Interview for the DSM-IV (SCID) (52), administered by trained raters of the SCID and confirmed by a doctoral-level clinician. All participants were clinically stable outpatients at the time of assessment. Inclusion criteria for participants at the Pittsburgh and Boston sites were: (1) between 18 and 45 years old; (2) current IQ ≥ 80, assessed by the WASI-II (53); and (3) fluent English speaker with the ability read at a sixth grade level or higher. Additional inclusion criteria for the participants with a psychotic disorder were: (1) a SZ or schizoaffective disorder diagnosis, verified using the SCID interview (54); (2) time since first psychotic symptoms of <10 years; and (3) clinically stabilized on antipsychotic medication. Inclusion criteria for psychotic disorder participants at the Belmont site were: (1) age 18–60 years; (2) diagnosis of SZ, schizoaffective disorder, or BD with psychotic features; and (3) clinically stable defined as no psychiatric hospitalization or medication change in the past month. Across sites exclusion criteria included: (1) significant neurological or medical disorders that might cause cognitive impairment (e.g., seizure disorder, traumatic brain injury); (2) persistent suicidal or homicidal behavior; (3) substance abuse or dependence present within the past 3 months; (4) any MRI contraindications; and (5) decisional incapacity requiring a guardian.

Neurotypical participants had never met criteria for any Axis I psychiatric disorder and had no history of head injury resulting in a loss of consciousness, seizure or neurological disorder. Table 1 summarizes the sample's demographic, clinical, and medication regimen information.

**Table 1:** Demographic and clinical information by group.

	**Probands (*n* = 81)**	**Controls (*n* = 45)**	**Statistical test**
Mean age (SD)	26.06 (7.19)	25.57 (5.93)	*t* = 0.411, *p* = 0.682
Sex	54 M, 27 F	22 M, 23 F	χ^2^ = 3.11, *p* = 0.078
Diagnosis	21 Bipolar Disorder, 51 Schizophrenia 9 Schizoaffective	–	N/A
Mean CPZE mg (SD)	261 (225)	–	N/A
Mean MSCEIT-ME	46.8 (13.0)	55.8 (9.4)	*t* = 4.48, *p* < 0.001
Mean FSIQ	109.8	110.7	*p = 0.671*

*CPZE, chlorpromazine equivalents; F, female; M, male; SD, standard deviation*.

### Cognitive Testing

The MATRICS Consensus Cognitive Battery (MCCB) was used to assess cognition (55, 56). This testing battery yields a cognitive composite score and 7 domain scores including processing speed, attention, working memory, verbal learning, visual learning, problem solving, and social cognition. In the MCCB, social cognition is assessed using the Mayer-Salovey-Caruso Emotional Intelligence Test (57) Managing Emotions branch (MSCEIT-ME). The MSCEIT-ME includes a series of vignettes. The vignettes are read aloud to participants as they follow along in their printed materials. Each vignette proposes a series of possible actions related to its scenario. The participants are asked to assess the effects each action would have on the actor's or other characters' mood states or behaviors. Responses follow a Likert-type scale. The MSCEIT-ME and MCCB scoring packages were used to calculate age and sex normed *T* scores.

Participants at the Boston and Pittsburg sites had full-scale (FSIQ) assessed using the Wechsler Abbreviated Scale of Intelligence (WASI). Participants at the McLean site had FSIQ and verbal IQ (VIQ) assessed using the North American Adult Reading Test (NAART).

### MRI Data Acquisition

Boston site: Data were acquired on 3T Siemens Trio (TIM upgrade) scanners using a standard head coil. The echoplanar imaging parameters were: repetition time, 3,000 ms; echo time, 30 ms; flip angle, 85°; 3 × 3 × 3-mm voxels; and 47 axial sections collected with interleaved acquisition and no gap. Structural data included a high-resolution T1 image. All participants underwent a resting-state fMRI run. Each functional run lasted 6.2 min (124 time points).

Pittsburgh site: Data were acquired on a 3T Siemens Verio scanner using a standard head coil. The echoplanar imaging parameters were: repetition time, 3,000 ms; echo time, 30 ms; flip angle, 85°; 3 × 3 × 3-mm voxels; and 45 axial sections collected with interleaved acquisition and no gap. Structural data included a high-resolution T1 image. The functional run lasted 6.2 min (124 time points).

McLean site (SZ): Data were acquired on 3T Siemens Trio (TIM upgrade) scanners using a standard head coil. The echoplanar imaging parameters were: repetition time, 3,000 ms; echo time, 30 ms; flip angle, 85°; 3 × 3 × 3-mm voxels; and 47 axial sections collected with interleaved acquisition and no gap. Structural data included a high-resolution T1 image. Each functional run lasted 6.2 min (124 time points) and the participants were given instructions to “remain still, stay awake, and keep your eyes open.”

McLean site (BP): Data were acquired on 3T Siemens Trio (TIM upgrade) scanners using a standard head coil. The echoplanar imaging parameters were: repetition time, 2,500 ms; echo time, 24 ms; flip angle, 82°; 3 × 3 × 3-mm voxels; and 42 axial sections collected with interleaved acquisition and no gap. Structural data included a high-resolution T1 image. Each resting-state functional run here lasted 10 min (240 time points) and the participants were given instructions to “remain still, stay awake, and keep your eyes open.”

### MRI Data Processing

MRI image preprocessing was performed as in presented in Ling et al. (51). DPABI image processing software was used to preprocess the imaging data (58). To minimize the scanner signal stabilization effects, the first images were omitted from all analysis (the first 4 images from 124 time point scans and first 10 images from 240 time point scans). We discarded scans with head motion that exceeded a 3 mm or 3° of maximum rotation threshold during the resting-state run. Functional and structural images were co-registered. Using the DARTEL technique (59), the structural images were normalized and segmented into gray, white and CSF partitions. Head motion effects were regressed out from the realigned data using a Friston 24-parameter model (60). CSF and white matter signals along with the global signal and the linear trend were regressed out. We incorporated the global signal regression because prior demonstration showed that combining it with volume-wise “scrubbing” for head “micromovements” is an effective method to remove motion artifacts (61). Following realignment, slice timing correction and co-registration, framewise displacement (FD) was calculated for all resting state volumes (62). All volumes within a scan that had a FD >0.2-mm were censored. Scans that required censoring half, or more, of their volumes were discarded. After nuisance covariate regression, the resultant data were band-pass filtered to select low frequency (0.01–0.08 Hz) signals. DARTEL normalized the filtered data into MNI space and then the data were smoothed by a Gaussian kernel of 8 mm^3^ full-width at half maximum (FWHM). Voxels contained within a group derived gray matter mask were used for further analyses.

After preprocessing, 126 participants, across all sites, remained in the study. 51 participants diagnosed with SZ, 9 with schizoaffective disorder, 21 with BD, and 45 neurotypical participants comprise our sample (Table 1).

### Functional Connectivity Analysis

#### Multivariate Distance Matrix Regression

**Figure 1:**
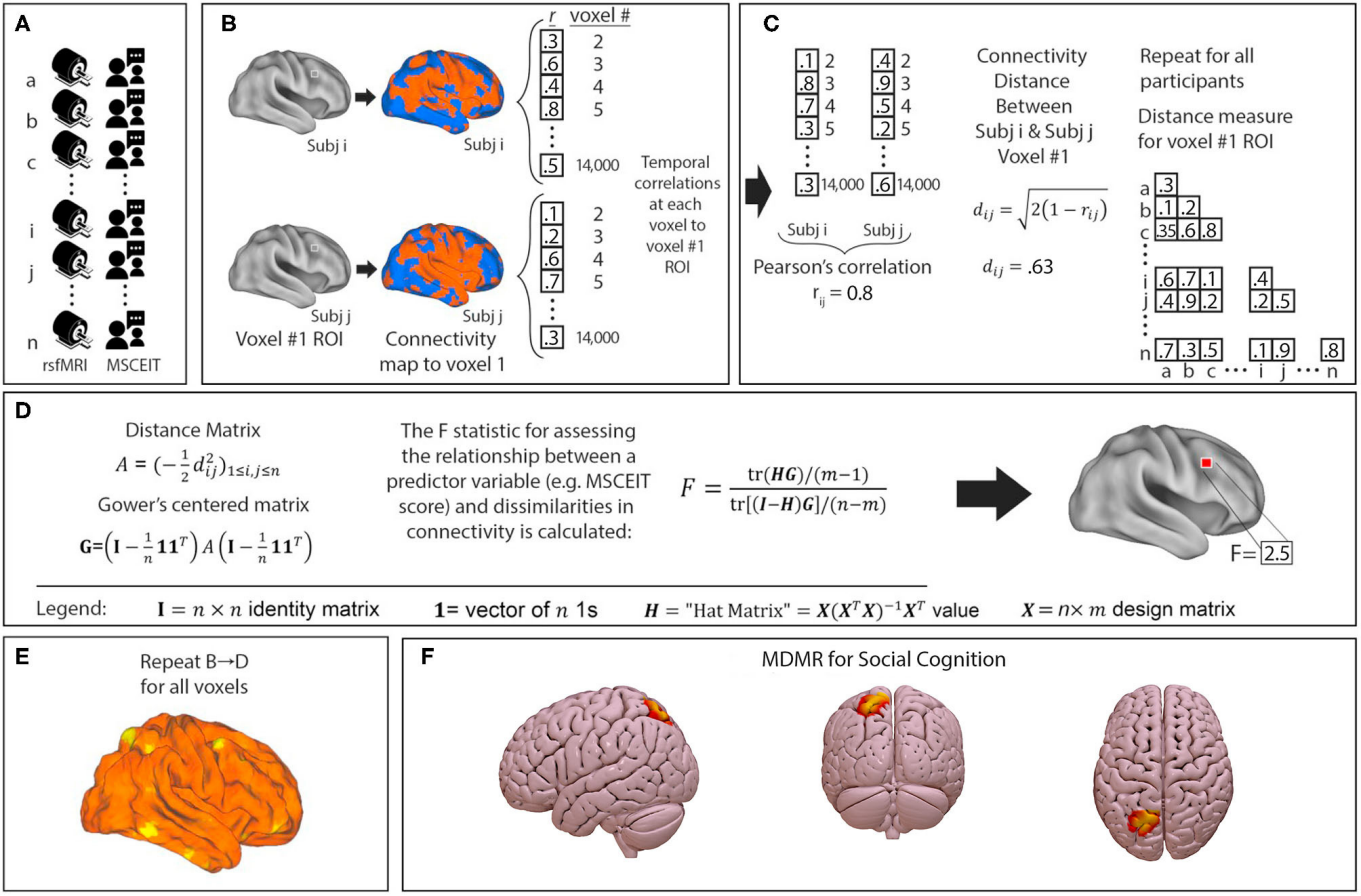
Multivariate distance matrix regression identifies left parietal connectivity as the strongest correlate of social cognitive ability in a trans-diagnostic sample. MDMR procedure: **(A)** rsfMRI and emotional intelligence testing are collected from each participant. **(B)** For each participant a functional connectivity map is generated to an individual voxel. **(C)** Voxelwise temporal correlations between participants are used to generate a Pearson's correlation *r* and a distance metric *d*. This is repeated for all participants to generate a matrix of between subject distances. **(D)** The distance matrix is centered and an ANOVA-like test is used to generate an *F*-statistic to assess the relationship between a predictor variable (MSCEIT-ME score) and dissimilarities in functional connectivity at that voxel. **(E)** This process is repeated for every voxel. This results in a whole brain map of how significantly functional connectivity is related to emotional intelligence. Permutation testing then identifies whole-brain significant clusters in connectivity-MSCEIT-ME relationships. **(F)** In our sample of 126 participants (*n* = 60 with schizophrenia or schizoaffective disorder, *n* = 21 with bipolar disorder with psychosis, and *n* = 45 neurotypical participants), we identified a single region in the left parietal lobule (centered at MNI coordinates *x* – 24 *y* – 69 *z* + 57) whose connectivity correlated significantly with emotional intelligence. In this image, connectivity is thresholded at a voxelwise level of *p* < 0.001 and extent threshold of *p* < 0.05.

We performed a connectome-wide association study using multivariate distance matrix regression (MDMR) as originally laid out in Shehzad et al. (63). In brief, MDMR tests every voxel to determine if whole-brain connectivity to that voxel is more similar in individuals with similar scores on an independent measure (MSCEIT-ME) than in individuals with dissimilar scores. As described (64–66), MDMR occurs in several stages: First, scan and MSCEIT-ME scores are collected from all participants (Figure 1A). Next, a seed-to-voxel connectivity map is generated for every participant. These maps are created by calculating the temporal Pearson's correlation coefficients between each voxel, using its BOLD signal time-course, and all other gray matter voxels (Figure 1B). Second, the temporal correlation coefficients for each voxel in the connectivity map are correlated with the values of corresponding voxels in the maps generated for the other participants. This Pearson's correlation coefficient, *r*, is a measure addressing how similar the whole-brain connectivity to a specific voxel is, for each voxel, between patients. This value is used to calculate between-subject distance (or dissimilarity) using the metric *d*_*ij*_ = $\sqrt{2\left(1-r_{ij}\right)}$ where *i* and *j* are two subjects and *r* is the correlation coefficient above (Figure 1C) (67). Third, we test the relationship between the independent variable of interest, here, MSCEIT-ME score, and the inter-subject distances in connectivity generated in the previous stage. Broadly speaking, this process consists of an ANOVA-like hypothesis test between a variable of interest and a matrix of distances. This method was originally named multivariate distance matrix regression by Zapala and Schork while they focused on associations between gene expression and related variables (67). Shezhad et al. then shifted their analytic focus, and used this framework to test the relationship between variables of interest and a matrix of distances, the matrix being similarity between-subject's whole-brain functional connectivity. This test first creates a distance matrix $A\;=\;{\left(-\frac{1}{2}d_{ij}^{2}\right)}_{1\leq i,j\leq n}$ among *n* participants where *d* = the between subject distance metric calculated above. Next, this matrix is used to create a Gower's centered matrix $G\;=\;\left(I-\frac{1}{n}11^{T}\right)A\left(I-\frac{1}{n}11^{T}\right)$, in which *n* is the number of participants, *I* is the *n* × *n* identity matrix, and 1 is a vector of *n* 1s. The *F* statistic for assessing the relationship between a predictor variable (e.g., MSCEIT-ME score) and dissimilarities in connectivity is calculated as follows: For *m* predictor variables, let ***X***be a *n* × *m* design matrix of predictor values, and let *H* = *X*(^*X*^^*T*^*X*)^−1^*X*^*T*^ be the associated *n* × *m* “hat” matrix.

$F\;=\;\frac{tr\left(HG\right)/\left(m-1\right)}{tr\left[\left(I-H\right)G\right]/\left(n-m\right)}$ (Figure 1D) (63). This process is repeated for every voxel. The result is a whole brain map showing how significant the relationship between MSCEIT-ME scores and functional connectivity is at every voxel (Figure 1E). From this generated map, ROIs for follow-up analysis are determined based on clusters of significant voxelwise *F*-statistics. To correct for multiple comparisons, a nonparametric permutation is calculated for voxels that exceed the significance threshold of *p* < 0.001 and clusters of such with an extent threshold of *p* < 0.05, with a null distribution calculated from 1,000 such permutations (Figure 1F). The voxelwise threshold was selected to maximize the replicability potential.

This MDMR analysis identifies anatomical regions where MSCEIT-ME score is significantly correlated with functional connectivity. Notably, this process does not consider spatial information about the voxels that give rise to between-individual distances. For example, two individuals may be very distant, or dissimilar, in the functional connectivity of a voxel in the precuneus. Such dissimilarity might be driven by differences in precuneus connectivity to the mPFC, temporal lobe, parietal lobe, or perhaps all three. MDMR, as implemented by Shehzad et al. (63), does not present this information. Visualizing this missing spatial information requires follow-on seed-based connectivity analysis. Shehzad et al. and others have defined this follow-on analysis as “*post-hoc*” testing to clarify that this alone, is not sufficient hypothesis testing nor an independent validation of the original MDMR finding (63–66). Following these prior manuscripts, we conducted the MDMR analysis to locate anatomical regions of interest where connectivity significantly correlated with MSCEIT-ME score and then performed follow-on seed-based connectivity analysis to detail the spatial distribution of these connectivity differences.

#### Seed Based Connectivity Analyses

We used DPABI for our seed-based connectivity analyses. This analysis extracted the BOLD signal time course in a 6 mm spherical ROI centered in the result of the MDMR (MNI *x* – 24 *y* – 69 *z* + 57). We then generated whole brain maps of *z*-transformed Pearson's correlation coefficients. We entered these maps into SPM12 (Statistical and Parametric Mapping, http://www.fil.ion.ucl.ac.uk/spm). Next, we regressed these maps against MSCEIT-ME scores. This process generated spatial maps that show how whole brain functional connectivity to the ROI varies with MSCEIT-ME score. We performed these analyses with sex, age, and scanner site as covariates to control for participant variables of non-interest.

In our sample, prescribed CPZE dosage was inversely correlated with MSCEIT-ME score (*r* = −0.445, *p* < 0.001). To control for possible medication regimen effects, this analysis was re-performed with the covariates above (age, scanner site, and sex) plus prescribed anti-psychotic dosage (in chlorpromazine equivalents, CPZE) as an additional covariate (Supplementary Figure 2).

#### ROI to ROI Analyses

To generate a scatter plot of the relationship between functional connectivity and MSCEIT score we extracted the BOLD signal time course between the MDMR centered ROI and the cerebellar cluster (thresholded at voxelwise *p* < 0.001).

Correlations between connectivity and MSCEIT and partial correlations with FSIQ or VIQ or CPZE as covariates were calculated using *r*.

#### Figure Generation

SurfIce was used to generate the projections of ROIs and T contrast maps onto cortical surfaces (www.nitrc.org/projects/surfice/).

## Results

### Functional Connectivity in the Superior Parietal Lobule Is Linked to Social Cognition

MDMR analysis performed across all 126 participants (51 SZ, 9 schizoaffective disorder, 21 BD with psychosis, 45 neurotypical) revealed a single region whose intrinsic functional connectivity correlated significantly with MSCEIT-ME social cognition scores. This identified a region in the left superior parietal lobule centered at MNI coordinates *x* – 24 *y* – 69 *z* + 57 (Figure 1F).

### Parietal–Cerebellar Connectivity Is Linked to Social Cognitive Ability

**Figure 2:**
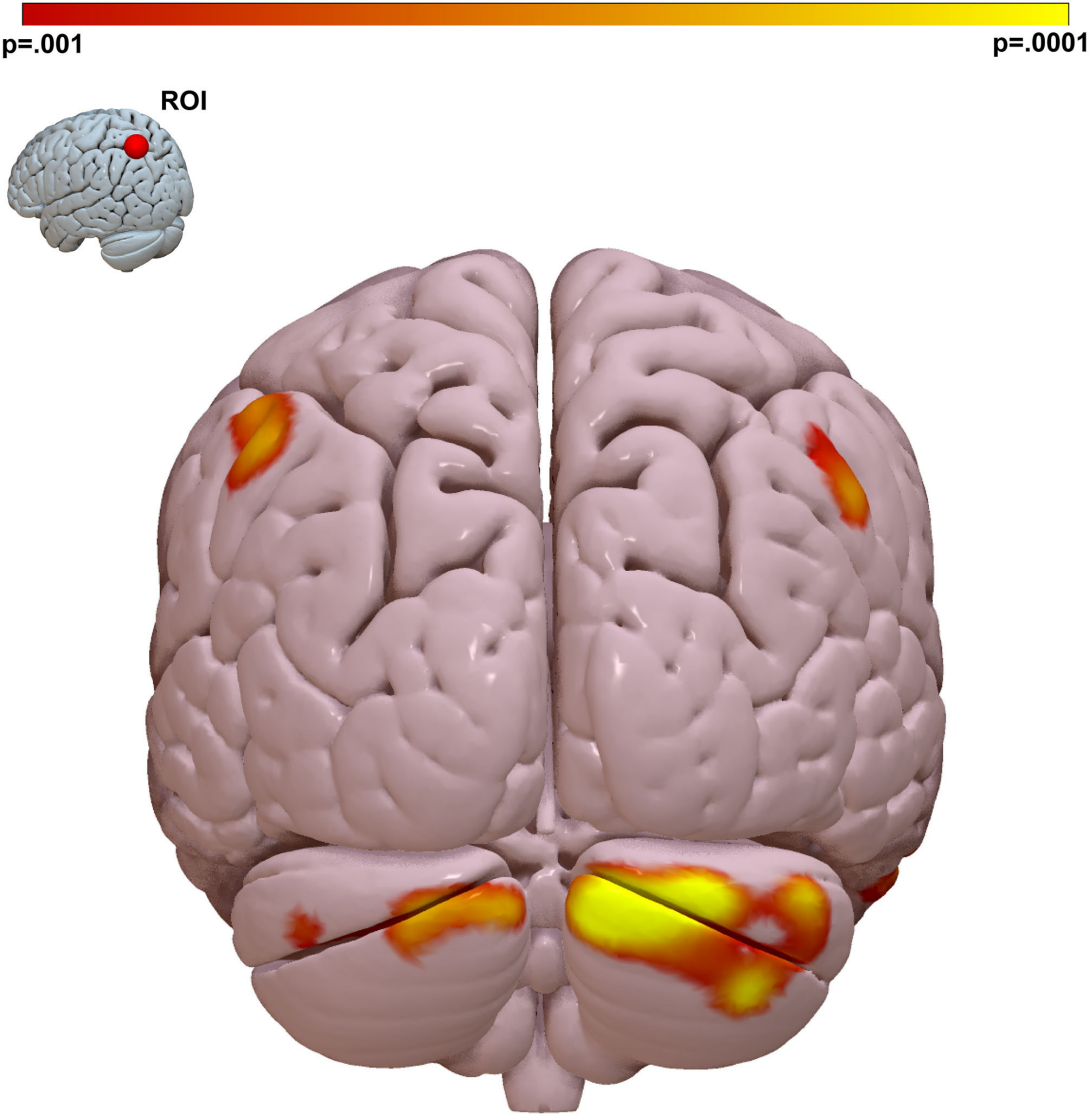
The strongest link between connectivity and social cognitive ability is a parietal Lobe-cerebellar Crus I, II circuit. We visualized the spatial distribution of connectivity that gave rise to the MDMR result in Figure 1. We placed a ROI in the left parietal region identified by MDMR and regressed connectivity to this region against MSCEIT-ME score. This identified the right cerebellar Crus I, II region as the region where functional connectivity correlates with social cognitive ability. Peak *T*-stat *T* = 4.99, *p* < 0.001, MNI *x* – 12, *y* – 90, *z* – 30. Cluster *k* = 695, *p*_FWE_ < 0.001. Color bar = voxel connectivity *p*-value.

We performed follow-on analysis using this parietal region in a seed-based connectivity analysis to determine the spatial distribution and directionality of connectivity that gave rise to this result. This analysis revealed that social cognition is positively correlated to functional connectivity between the left parietal lobe and other regions of the DMN including DMN nodes in both bilateral parietal lobes and bilateral cerebellum. This relationship was observed maximally between left superior parietal lobe and the Crus I, II region of the cerebellum (Figure 2 and Supplementary Figure 1).

In our sample participants with a psychotic disorder demonstrated social cognitive ability a full standard deviation below the neurotypical participants (Table 1). When we examined individual diagnostic groups, we observed that the relationship between connectivity and cognition was similar for all groups: Neurotypical participants: *r* = 0.434, *p* = 0.003; BD participants: *r* = 0.448, *p* = 0.042. SZ/schizoaffective participants: *r* = 0.394, *p* = 0.002. Comparing the strength of correlation between groups did not reveal significant differences between neurotypical and BD groups (*p* = 0.952), between neurotypical and SZ /schizoaffective groups (*p* = 0.810), or between bipolar and SZ/schizoaffective groups (*p* = 0.810).

To isolate social cognition specific effects we calculated the partial correlation between parietal–cerebellar connectivity and MSCEIT with estimated IQ regressed out as a covariate. We continued to observe the same strong correlation between connectivity and MSCEIT score *r* = 0.410, *p* < 0.001. A subset of the participants (*n* = 56) also had verbal IQ estimated by NAART. In this subset of participants, the partial correlation of connectivity with MSCEIT score with VIQ as a covariate remained highly significant *r* = 0.555, *p* < 0.001.

As reported above (section “Seed Based Connectivity Analyses”) we observed a significant inverse correlation between prescribed CPZE dosage and MSCEIT score. We observed the same cerebellar–parietal connectivity-cognition relationship in all diagnostic subgroups (including neurotypical participants not taking antipsychotics) making it unlikely that observed connectivity is caused by medication effects. We calculated the partial correlation between parietal–cerebellar connectivity and MSCEIT with CPZE regressed out as a covariate. We continued to observe the same strong correlation between connectivity and MSCEIT score *r* = 0.364, *p* < 0.001. We also regressed maps of connectivity to the parietal ROI against MSCEIT score with CPZE as an additional covariate (in addition to age, sex, and site) and continued to identify a significant correlation to the right cerebellum (Supplementary Figure 2), albeit at a lower voxelwise significance threshold (*p* < 0.005).

## Discussion

We present the results of our efforts to identify brain circuit correlates of social cognition. Our approach included a trans-diagnostic cohort of neurotypical adults and participants with psychotic disorders. As predicted, participants with psychosis exhibited significant impairment in social cognition compared to controls. We then used a fully data driven analysis of task-free connectivity at the individual voxel level to find the strongest correlates of social cognitive ability. This approach determined that functional connectivity between left superior parietal cortex and other nodes of the DMN are positively correlated with social cognitive ability. A link between cognition and SPL connectivity was observed in bilateral nodes of the DMN but there was a laterality to the strongest result observed. Specifically, the strongest relationship between functional connectivity and social cognitive ability was observed in a circuit between right cerebellar Crus I, II and left superior parietal cortex. The relationship between cognition and connectivity at those nodes was trans-diagnostic and observed in both neurotypical participants as well as those with psychotic disorders, despite the participants with psychotic disorders performing, on average, a full standard deviation worse than neurotypical adults. This is consistent with a model in which cerebellar–parietal connectivity mediates the relationship between diagnosis and social cognitive ability. This observation is in line with a recent consensus report highlighting the role of the cerebellum in social cognition (68). Interestingly, a recent large study in SZ found robust reductions in cerebellar gray matter volume with the strongest effects in regions that were functionally connected with frontoparietal cortical regions (69) suggesting that not only is cerebellar–parietal connectivity linked to social cognitive processing, but that it is strongly associated with abnormalities in psychosis.

Historically, hypothesis driven neuroimaging has focused on the prefrontal cortex in studies involving complex cognition such as working memory and social reasoning. How can our result be reconciled with the extant literature? Strikingly, this discovery is entirely consistent with prior findings in both human disorders of social cognition (e.g., autism) and in murine models. Case-control studies in ASD have consistently identified abnormalities in the Crus I, II region of the cerebellum but the functional consequence of this finding had been unclear. More recently, through innovative experiments, Stoodley et al. demonstrated that cerebellar neuromodulation in humans can manipulate cerebellar–parietal connectivity. Those investigators were able to extend this result by demonstrating with direct recording that right Crus I Purkinje neurons modulate activity in mouse parietal association cortex (48). Both that study and a subsequent paper demonstrated a critical role for Crus I in social preference in mice (48, 70).

Here we expand on these studies in two critical ways: First, while prior studies demonstrated R Crus I of the cerebellum can modulate parietal activity in humans, we demonstrate that communication between cerebellum and parietal lobe is directly related to human social cognitive ability. Second, we demonstrate that this circuit can account for individual variance in social cognitive ability in disorders of impaired social cognition (e.g., SZ) as well as in neurotypical humans.

Of particular note, we arrive at this circuit using a whole-brain, data-driven analysis, i.e., without limiting ourselves *a priori* to these candidate regions. This circuit is identified as *the strongest* link to social cognition in our sample. Thus, we observe a convergence of results from independent data in humans and mice identifying a trans-diagnostic and *trans-species* cerebellar-cortical circuit with evidence of a *causal* link to social cognitive ability.

We suggest that this convergence of results is also a product of the analytic approach used here. Specifically: The participants of this sample represented a spectrum of social cognitive ability. That variance is presumably linked to a variety of underlying causes, i.e., some participants had social cognitive deficits linked to a primarily genetic disorder (SZ) and other participants whose abilities represent normal population variation not linked to the genetic causes of SZ. In finding a common brain substrate for social cognitive ability irrespective of etiology, we propose that this connectivity-cognition link may be common pathway mediating social cognitive ability. That is, a circuit casually linked to cognition rather than epiphenomena linked to disease severity.

The results presented in these studies link circuit connectivity to social cognition as measured by tests in laboratory conditions. However, data suggest that there may be real-world outcomes linked to this circuit as well. Smith et al. analyzed human connectome project data using canonical correlation analysis to link a wide range of tests and life experiences to functional connectivity (71). This analysis revealed a broad range of outcomes organized along a “positive–negative” axis (e.g., life satisfaction as a positive outcome, and THC use as a negative outcome). Strikingly, the strongest brain link to this axis was functional connectivity between cerebellar Crus I, II and parietal lobule.

**Figure 3:**
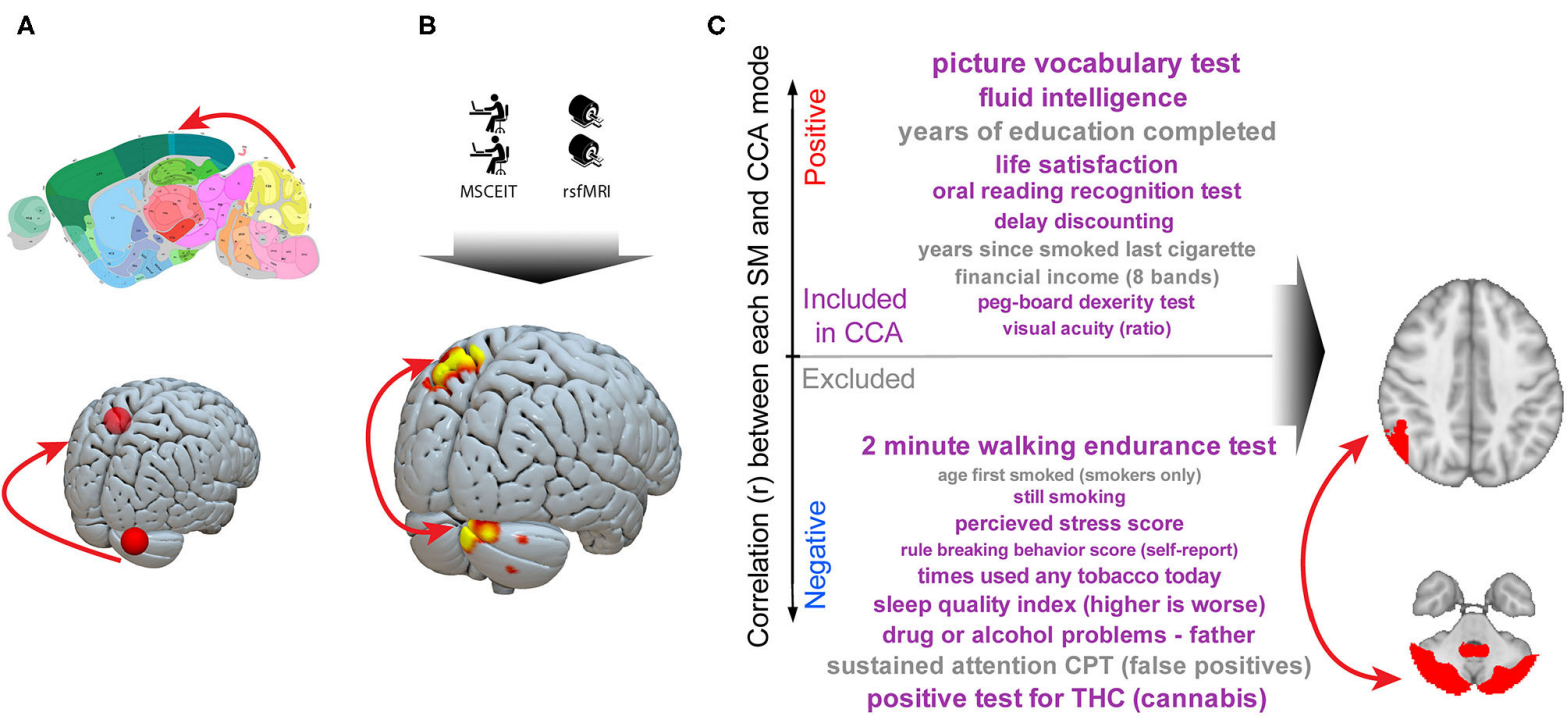
A cerebellar–parietal circuit is causally linked to social cognition both trans-diagnostically and trans-species. A series of murine and human experiments converge on a shared circuit causally linked to social cognition. **(A)** Imaging studies reliably identify cerebellar right Crus abnormalities in autism. Neuromodulation experiments in humans identify a circuit linking right Crus to the left parietal lobe and murine studies demonstrate this circuit is critical to normal social interaction (48). **(B)** We observe that, in a trans-diagnostic sample, connectivity between right cerebellar Crus and left parietal lobe connectivity is directly linked to social cognitive ability. **(C)** In a large (*n* = 461) dataset, connectivity in this cerebellar Crus–parietal circuit was the strongest link between a broad array of outcomes along a “positive–negative axis” (71). Taken together, these data are consistent with a critical role for a cerebellar–cortical circuit in complex social cognition. Murine image from the Allen Mouse Brain Atlas.

These results in murine behavioral tests, human social cognitive test performance, and real-world outcomes are independently derived but all converge on the same consensus cerebellar–parietal circuit. This allows the construction of an empirically derived model in which social cognition is critically dependent on this cerebellar-cortical circuit function (Figure 3).

Prior evidence for a cerebellar role in organized cognition has come from lesions and correlational imaging studies (72). A wealth of recent murine studies has demonstrated a critical role for the cerebellum in multiple aspects of cognition (73–77). In this model we add social cognition to the growing list of cognitive domains dependent on cerebellar computation. In particular, the MSCEIT-ME branch requires participants to listen to vignettes and make predictions about the emotional or social consequences of various possible actions; the association between performance on this task and the identified cerebellar–parietal circuit is consistent with findings that the cerebellum, and specifically Crus I and II, plays a role in social cognition via social prediction (68).

What is the relevance of this result to disease? The evidence presented here link social cognitive impairment in psychotic disorders to this circuit. We previously demonstrated, in an independent data set, that hypoconnectivity in a cerebellar–Dorso-Lateral Pre-Frontal Cortex (DLPFC) circuit is *causally* linked to negative symptoms (e.g., apathy) in SZ (78). The cerebellar node of that circuit is the *same* Crus I, II region we link to social cognition in the current study. Specifically: connectivity between this Crus I, II region and different cortical regions is linked to different deficits in SZ: the left parietal lobe for social cognition and the right DLPFC for negative symptoms. This allows a mechanistic model for the co-occurrence of these deficits in SZ: Distinct deficits result from dysconnectivity in specific circuits, but all of these circuits have a shared node in the cerebellum.

Importantly, these findings have implications for targeted interventions to improve social cognitive functioning in people across diagnostic boundaries. Our study using transcranial magnetic stimulation (TMS) to target a cerebellar-cortical circuit associated with negative symptoms in psychosis found that neuromodulation at the cerebellar site was associated with both increased connectivity *and* reduction in negative symptoms (78). Our findings, together with others [e.g., (48)], identify a potential neural target for improving social cognition that may be both modifiable and associated with downstream pro-cognitive effects.

One limitation of our study was the use of a single test of social cognitive ability. The MSCEIT-ME test included in the MATRICS consensus cognitive battery was designed to measure a specific aspect of social cognition, higher-order emotional reasoning regarding emotion management and regulation, and does not measure other social cognitive domains such as theory of mind or emotion perception. That said, the managing emotions domain of MSCEIT-ME is linked to real world functional outcomes (79) and the broad adoption of the MATRICS allowed consolidation of samples from across multiple sites (80). The MSCEIT-ME branch was also the only branch of the MSCEIT in which people with psychosis continued to differ from controls after controlling for general cognitive ability (81), suggesting that it is tapping emotional intelligence in a way that is at least partially distinct from general cognitive skills. Additionally, the MSCEIT-ME was among the MSCEIT branches most strongly associated with brain volume measures in people with SZ and related disorders (82). However, associations between this circuit and other domains of social and emotional processing remain to be determined. Another limitation is that we did not have uniform data on social or other functional outcomes across the sample and were therefore unable to evaluate effects of our findings on real-world social functioning.

Despite these limitations, the convergence of results linking social cognition to a cerebellar–parietal circuit (Figure 3) argues (1) dysfunction in this circuit is linked to social cognition trans-diagnostically in psychotic disorders and (2) at the circuit level these deficits lie along a continuum with variation in social cognitive ability in a neurotypical population. Future studies can determine if individual variation in social cognitive ability in ASDs covaries with cerebellar–parietal connectivity. Evidence from murine experiments are consistent with a *causal* relationship between this circuit and social cognition. From a basic science perspective, the convergence of human and murine findings suggest that this circuit is a valid candidate for modeling how circuit dysfunction gives rise to social cognitive phenotypes in psychiatric disorders. Therapeutically, prior work has established that this circuit can be manipulated non-invasively (48) making it a promising candidate target for interventions designed to ameliorate social cognitive deficits.

## Data Availability Statement

The raw data supporting the conclusions of this article will be made available by the authors, without undue reservation.

## Ethics Statement

These studies were reviewed and approved by the University of Pittsburgh, BIDMC, and McLean Hospital IRBs.

## Author Contributions

RB was involved in all aspects of this project including study design, statistical analyses, development of tables and figures, and drafting of the manuscript. AB was involved in statistical analyses, development of figures, and drafting of the results, and methods. MN was involved in data collection and analysis. SE was involved in development of studies in which the data were collected. RM-G was involved in design and interpretation of the cognitive data. MK was involved in development of studies in which the data were collected and the analysis plan. KL was involved in development of studies in which the data were collected, development of the present project, and drafting of the manuscript. All authors reviewed and approved the final version.

## Conflict of Interest

The authors declare that the research was conducted in the absence of any commercial or financial relationships that could be construed as a potential conflict of interest.
