# Supplementary material for: Cerebellar-Cortical Connectivity Is Linked to Social Cognition Trans-Diagnostically
Source: Front Psychiatry. 2020 Nov 4;11:573002. doi: 10.3389/fpsyt.2020.573002 (PMC7672118; doi:10.3389/fpsyt.2020.573002)
Supplement: Supplementary file 1 [file Data_Sheet_1.docx]

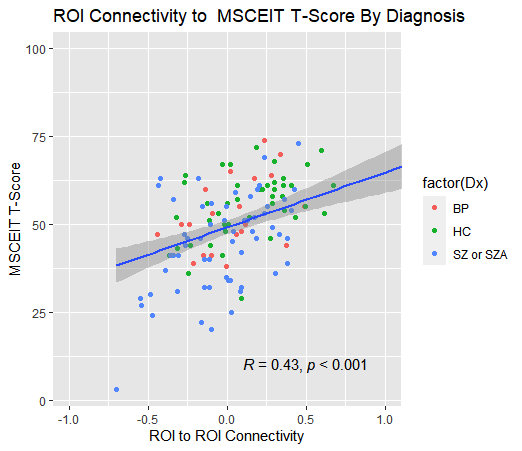


**Supplemental Figure 1.** The relationship between social cognition score and functional connectivity. Connectivity values represent z-transformed Pearson’s correlation coefficients between the left parietal ROI and the cerebellum region visualized in Figure 2.

**
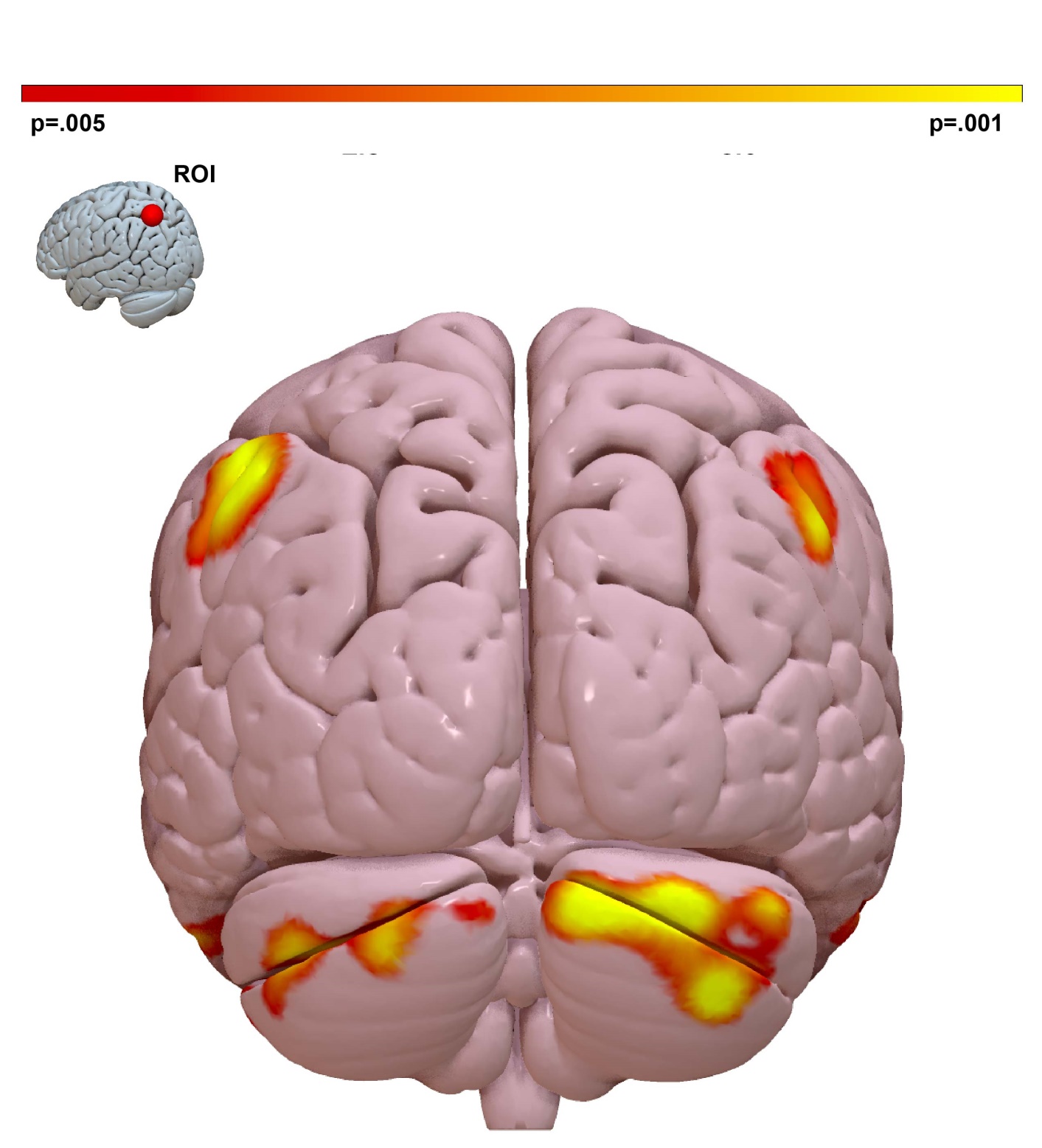
**

**Supplemental Figure 2.** Functional connectivity of the parietal ROI regressed against MSCEIT score analysis with site (sequence), age, sex, CPZE as covariates. This is the same analysis shown in Figure 2 but with the addition of CPZE as a covariate in the 125 participants with CPZE dosage recorded. Thresholded voxelwise p<.005. Peak T-stat T=4.15, p<.001, MNI x27, y-87, z-27. Cluster k=833, P_FWE_=.004
